# Radius and Orientation Measurement for Cylindrical Objects by a Light Section Sensor

**DOI:** 10.3390/s16111981

**Published:** 2016-11-23

**Authors:** Youdong Chen, Chongxu Liu

**Affiliations:** School of Mechanical Engineering & Automation, Beihang University, Beijing 100191, China; liucx@buaa.edu.cn

**Keywords:** ellipse recognition, rough identification, cylindrical objects

## Abstract

In this paper, an efficient method based on a light section sensor is presented for measuring cylindrical objects’ radii and orientations in a robotic application. By this method, the cylindrical objects can be measured under some special conditions, such as when the cylindrical objects are welded with others, or in the presence of interferences. Firstly, the measurement data are roughly identified and accurately screened to effectively recognize ellipses. Secondly, the data are smoothed and homogenized to eliminate the effect of laser line loss or jump and reduce the influence of the inhomogeneity of measurement data on the ellipse fitting to a minimum. Finally, the ellipse fitting is carried out to obtain the radii and orientations of the cylindrical objects. Measuring experiments and results demonstrate the effective of the proposed radius and orientation measurement method for cylindrical object.

## 1. Introduction

Cylindrical objects are widely used in industry. When cylindrical objects (for example, pipes, thin rods) are processed such as by one side welding or single punch, warps and deformations always occur. In order to eliminate the effects of the warps and deformations, the radii and orientations of the cylindrical objects must be measured before processing.

There are many ways to measure the radius and orientation of cylindrical objects. They can be generally classified into contact and non-contact measurements. There are lots of methods for non-contact measurement, such as electromagnetic induction, visual and laser imaging measurement. Among them, the visual measurement methods and laser measurement methods are widely used for cylindrical object measurement purposes.

For the visual measurement of cylindrical objects, a 3D vision system using highlight patterns formed by specular reflection on the object surface has been proposed in [1]. Two light sources were employed at once for the reduction of the processing time, which can only measure one cylindrical object at a time. Wang et al. [2] presented a high precision detection of cylindrical objects using one camera, which can only be used to measure the diameter of one small size cylinders at a time. An efficient relative pose estimation method based on multi-microscopic vision was presented in [3] for the alignment of long cylindrical components in six DOF (degree of freedom) of 3D space. In [4], an approach to applications of object detection and pose estimation from noisy RGB-D sensor (a kind of sensor which can capture and depth information) was presented. It can also be used to determine incomplete object poses, including those of symmetrical objects. Oleari et al. [5] presented the design and experimental evaluation of an embedded vision system for underwater object detection. The design approach has focused on a low power budget, low cost, and inevitably low performance embedded system. The system has proven thermally stable and capable of guaranteeing a level of autonomy of at least two hours of video acquisition.

For the laser measurement of cylindrical objects, an accurate long-distance position measurement system for cylindrical objects using laser range finders was proposed in [6]. This research aimed to estimate accurate center positions of the objects by applying the least squares method or maximum likelihood estimation to their contours based on the object shape information. An approach using a mobile robot equipped with a laser range finder to automatically measure the coordinates of cylindrical objects was presented in [7]. In this method, the near-optimal solution is obtained using the proposed adaptive distance method (ADM) and an extended Kalman filter (EKF). Richtsfeld et al. [8] presented a system with a fixed robot arm and a scanning unit, which is able to detect and grasp the given cylindrical objects with cluttered adjacent objects in soft real-time. It is based on scanning the objects on the table with a rotating laser range scanner, focused on the execution of subsequent path planning and grasping motions. Obviously, the systems in [6,7,8] only estimated the centers and the diameters of the cylindrical objects, but they were unable to measure the orientation. In addition, [9,10,11,12] reported a series of applications of laser telemetry measurement of cylindrical objects with a basically similar system composed of a multi-planar laser transmitter and a receiving camera. The calculation are based on the property that a circle is entirely defined by three of its tangents, which they called it “three-tangent method”, which can obtain the radius and orientation of one cylindrical object at a time. 

In addition to the above two major categories of measurement methods for cylindrical objects, [13] presented an approach for determining the diameter of cylindrical objects based on the available two triangles formation technique, which was purposely proposed for use in small-scaled unmanned aerial vehicles. A low-cost solution based on an IR sensor array was presented in [14] for the roughly determination of the characteristic properties of cylindrical objects like size, location, trajectory and so on. It is based on the application of multiple infrared sensors for the extraction of the surface features of cylindrical objects. Some shockwave-scattering simulations of backscattered signals were performed, where a broadband shockwave was used as the incident wave [15]. The radius and thickness of a cylindrical target was estimated by analyzing the echo signal in the time domain and the echo spectrum in the frequency domain. A general equation for the hyperbolae which result from buried cylinders was presented in [16] which allowed for measuring cylinders of arbitrary radius, resulting in a more accurate estimation of the buried cylinders’ depth and the surrounding medium’s relative permittivity, in addition to the radius information. It is achieved by subjecting the radargrams to a series of image processing stages followed by a curve-fitting procedure that was specifically developed for hyperbolae. Although these methods can measure the diameters and orientation successfully, they can’t be used in actual situations under some special conditions, for example, when the cylindrical objects are welded with others, or interferences, etc. 

Especially for the estimation of cylindrical object deformation, a pose-sensing system for soft robot arms was introduced in [17]. It integrates a set of macro-bend stretch sensors, which consist of optical fibers. The macro-bend sensor is based on the notion that bending an optical fiber modulates the intensity of the light transmitted through the fiber, and it is capable of measuring bending, elongation and compression in soft continuum robots and is also applicable to wearable sensing technologies. Similar to this, [18] proposed a non-linear approach to predicting the robot arm posture, by training a feed-forward neural network with a series of structured pressures values applied to the arm’s actuators. The ability to estimate pose is based on data from a custom fiber-optic bending sensor and accompanying model which is developed across a set of seven different experiments. Obviously, these two methods are different from the research concerns of this paper.

The motivation of this study was to develop an efficient approach for measuring the radius and orientation of a cylindrical object subject to interference. The presented method for measuring the radii and orientations of cylindrical objects is employed in a light section sensor. A light section sensor, which is a kind of compact laser sensor, was used to measure the profile of cylindrical objects at the cross section. The measurement data are fitted to ellipses by using a least squares method based on absolute Euclidean distance [19]. The main contributions of this paper are:

- A new method for non-contact measurement of the radius and orientation of cylindrical object is presented. This method can measure the radius and orientation with interference.
- This method can obtain the radii and orientations of several cylindrical objects in one measurement.

The rest of this paper is organized as follows: Section 2 gives an introduction of the light section sensor and the calculation of the radius and orientation. Section 3 details the measurement process and data processing method. Section 4 shows experiments and result analyses. Finally, this paper is concluded in Section 5.

## 2. Principle

### 2.1. Light Section Sensor

**Figure 1:**
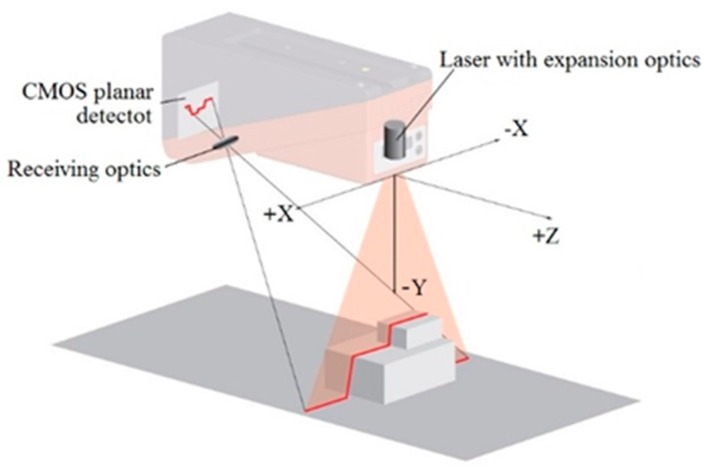
Method of detection.

A light section sensor is a kind of laser sensor that works according to the triangulation principle. Using transmission optics, a laser beam is expanded to a line aimed at a measurement object, as shown in Figure 1. The laser lines reflected by the measurement object are detected by the receiver located behind the laser transmitter. The laser transmitter is composed of the receiving optics and the CMOS chip, processes and transforms the reflecting laser lines into distance data in internal calculus, achieves surface profile.

Measurement data are a series of discrete points, which have *X* and *Y* coordinates. Obviously, the light section sensor can only measure the 2D data in one operation. The 3D data can then be obtained from the process control or by calculation.

### 2.2. Measurement by the Light Section Sensor

#### 2.2.1. Measurement Scheme

**Figure 2:**
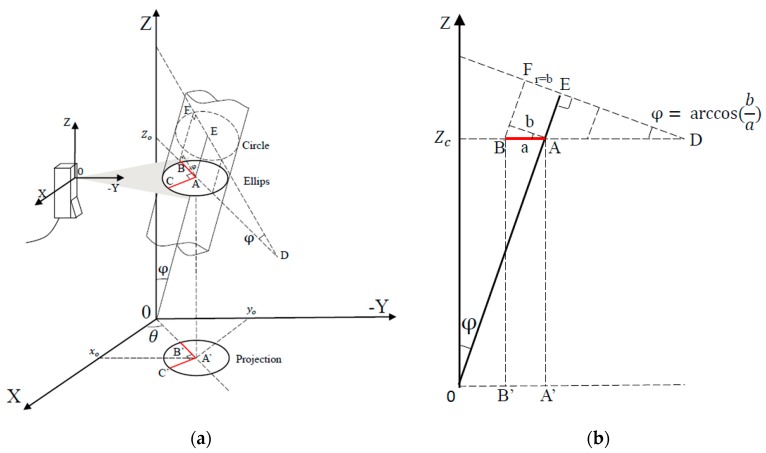
Measurement by the light section sensor: (**a**) Schematic diagram of the overall measurement; (**b**) View along CA direction.

Supposing the cylindrical object under test is tilted on the horizontal plane, it’s illuminated by a laser plane paralleled to the horizontal plane, thus creating a section containing an ellipse arc, shown in Figure 2a.

In the case of a tilted cylindrical object, because of its general rotating body characteristics, it is not necessary to consider the angle of rotation around the axis. The orientation can be determined by two linear independent parameters, the angle *φ* between the cylindrical object axis and the *OZ* axis, the angle *θ* between the projection of the major axis of the ellipse arc and the horizontal axis, shown in Figure 2a.

The ellipse arc determined by three points A, B, C is on the laser plane of sensor, which is parallel to the *XOY* plane. The point A (*x_o_*, *y_o_*, *z_o_*) is the center of the ellipse. The segments AB and AC are the major semi-axis and the minor semi-axis, respectively. The lengths of AB and AC are *a* and *b*. AE is the axis of the cylindrical object. A’, B’ and C’ is the projection of A, B, C on the *XOY* plane. The angle between the major axis direction and the *OX* direction is *θ*. That is the angle between A’B’ and the axis *OX*. The angle between AE and axis *OZ* is *φ*. BF is one of the generatrices of the cylindrical object. The terms *a*, *b* and *θ* can be calculated by fitting the measurement data into an ellipse.

#### 2.2.2. Radius Calculation

The value of AC is the value of the actual radius of the cylindrical object. The actual radius of the cylindrical object equals the minor semi-axis of the ellipse. That is *r* = *b*

**Proof.**  ∵ OZ⊥plane ABC, ∴ OZ⊥AC; ∵ AC⊥AB and AC⊥OZ, ∴ AC⊥plane ABO; ∵ AE⊂ plane ABO, ∴ AE⊥AC, ∴ AC is one of the radii of the cylindrical object. □

#### 2.2.3. Orientation Calculation

Because the cylindrical object is rotationally symmetric, the two linear independent degrees of freedom *θ* and *φ* can determine the orientation of the cylindrical object in space.

As shown in Figure 2a, *θ* is the angle between X axis and the projection of the cylindrical object axis in the XOY plane. As shown in Figure 2b, according to the trigonometric relations, *φ* can be obtained by the arccosine value of the ratio EF/AB, and the ratio equals to the ratio of the radius *r* (equals to the minor semi-axis, *b*) and the major semi-axis *a*. That is *φ* = *cos^−1^(b/a)*.

### 2.3. Fitting Method

The ellipse arc paralleled to the horizontal plane. The equation for the ellipse arc can be simplified as:

(1) $$\frac{{\left(\left(x_{i}-x_{o}\right)\sin\;\theta -\left(y_{i}-y_{o}\right)\cos\;\theta \right)}^{2}}{a^{2}}+\frac{{\left(\left(x_{i}-x_{o}\right)\cos\;\theta +\left(y_{i}-y_{o}\right)\sin\;\theta \right)}^{2}}{b^{2}}=1$$

where (*x_i_*, *y_i_*) are the boundary points.

**Figure 3:**
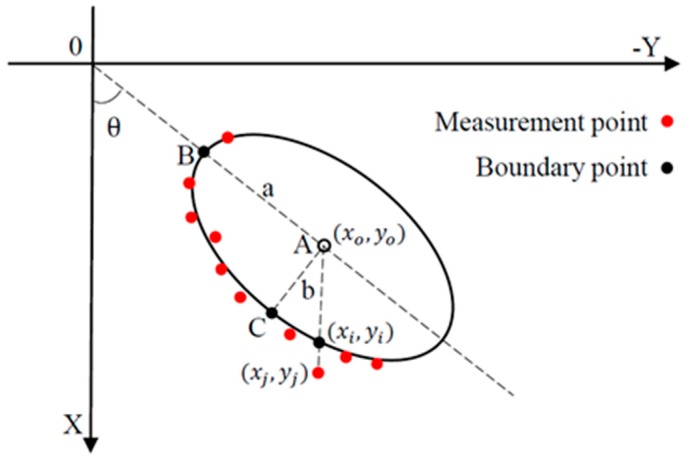
Absolute Euclidean distance between points and fitting ellipse.

The measured points are a series of discrete points, shown in Figure 3. In order to identify the ellipse, we adopted a least squares fitting method based on the absolute Euclidean distance.

The point (*x_i_*, *y_i_*) is on the ellipse, and the corresponding measurement point is the point (*x_j_*, *y_j_*). The distance between them can be expressed by:

(2) $$r_{j}=\left|\sqrt{{\left(x_{j}-x_{o}\right)}^{2}+{\left(y_{j}-y_{o}\right)}^{2}}-\sqrt{{\left(x_{i}-x_{o}\right)}^{2}+{\left(y_{i}-y_{o}\right)}^{2}}\right|$$

Since the point (*x_i_*, *y_i_*) is on the ellipse, it satisfies Equation (1). Because the points (*x_j_*, *y_j_*), (*x_i_*, *y_i_*) and (*x_o_*, *y_o_*) are on the same line, we have:

(3) $$\frac{y_{j}-y_{o}}{x_{j}-x_{o}}=\frac{y_{i}-y_{o}}{x_{i}-x_{o}}$$

Taking Equations (1) and (3) into Equation (2), eliminating the latter half of Equation (2) which contains (*x_i_*, *y_i_*), the *r_j_* can be rewritten as:

(4) $$r_{j}=\left|\sqrt{{\left(x_{j}-x_{o}\right)}^{2}+{\left(y_{j}-y_{o}\right)}^{2}}-\sqrt{\frac{{\left(x_{j}-x_{o}\right)}^{2}+{\left(y_{j}-y_{o}\right)}^{2}}{{\left(x_{j}-x_{o}\right)}^{2}}\frac{a^{2}b^{2}}{a^{2}{\left(\frac{y_{j}-y_{o}}{x_{j}-x_{o}}\sin\;\theta +\cos\;\theta \right)}^{2}+b^{2}{\left(\sin\;\theta -\frac{y_{j}-y_{o}}{x_{j}-x_{o}}\cos\;\theta \right)}^{2}}}\right|$$

In order to get the fitting ellipse, we employ the least squares method:

(5) $$min\left(J\right),\;\mathrm{subject}\;\mathrm{to}\;J=\overset{n}{\underset{j=1}{\sum }}r_{j}^{2}$$

The terms *a* and *b* are initialized by the radius of the cylindrical object. The initial value of *θ* is set to zero. The point with the maximum *y* value is recorded as (*x_ymax_*, *y_ymax_*). The *x_ymax_* is assigned to the initial value of *x_o_*. The value that the *y_ymax_* minus the cylindrical object’s radius is assigned to the *y_o_* initial value. To obtain the minimum value of the *J*, Equation (4) is calculated iteratively. After the minimum value of the *J* is obtained, the corresponding (*x_o_*, *y_o_*), *a*, *b* and *θ* are obtained.

## 3. Measurement Procedure

### 3.1. Measurement Process

**Figure 4:**
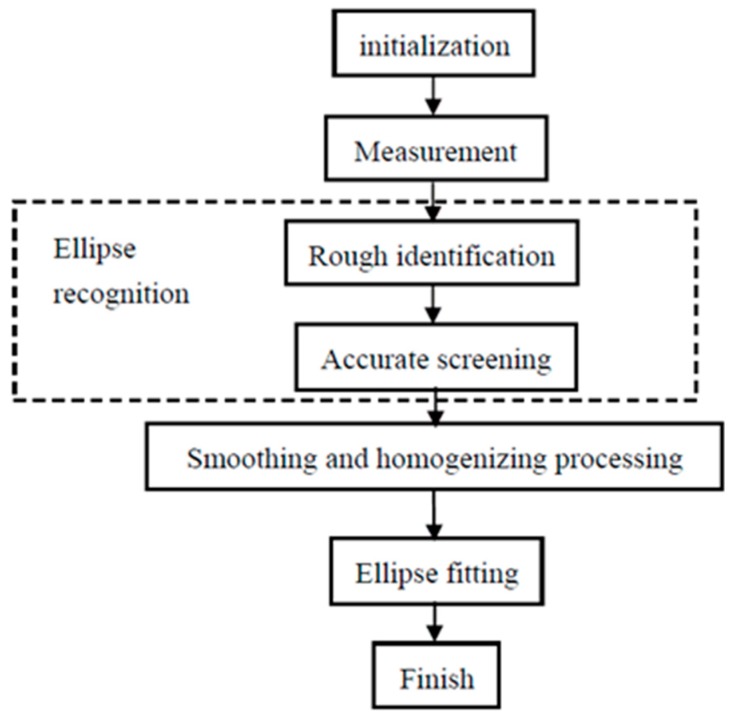
The flowchart of the measurements.

The measurement of the radius and orientation of the cylindrical object includes ellipse recognition, smoothing and homogenizing, and ellipse fitting. The whole measurement process is summarized in Figure 4. First of all in the procedure, the initial poses of the light section sensor and cylindrical object are manually adjusted into place. The ellipse fitting fits the measurement points onto an ellipse to get the elliptic parameters, then calculates the radius and orientation.

### 3.2. Ellipse Recognition

Ellipse recognition includes rough identification and accurate screening. In a practical environment, the measurement objects are not only cylindrical objects, but also other shaped ones, such as pipes that are welded side by side on a flat bar. Because the least squares method minimizes the quadratic sum of the global data’s errors, the points in the transition part between the ellipse contour and others have a great influence on the overall fitting result. In order to accurately fit the ellipse and precisely calculate the radius and orientation, it’s necessary to exclude the non-ellipse arc points as much as possible.

**Figure 5:**
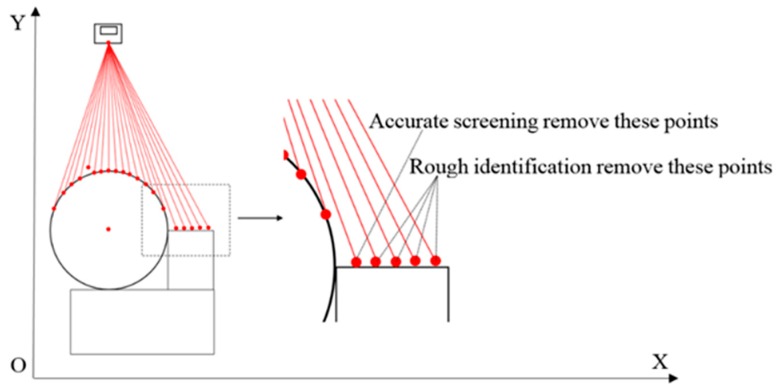
Ellipse recognition.

The rough identification judges whether a point belongs to an ellipse, as shown in Figure 5. If the point was relatively close to the ellipse and does not belong to the ellipse, the rough identification would no longer be effective. For example, considering cylindrical objects welded together, because the weld seams usually have circular contours, their measurement points cannot be accurately identified by rough identification only. An accurate screening can remove these points.

#### 3.2.1. Rough Identification

**Figure 6:**
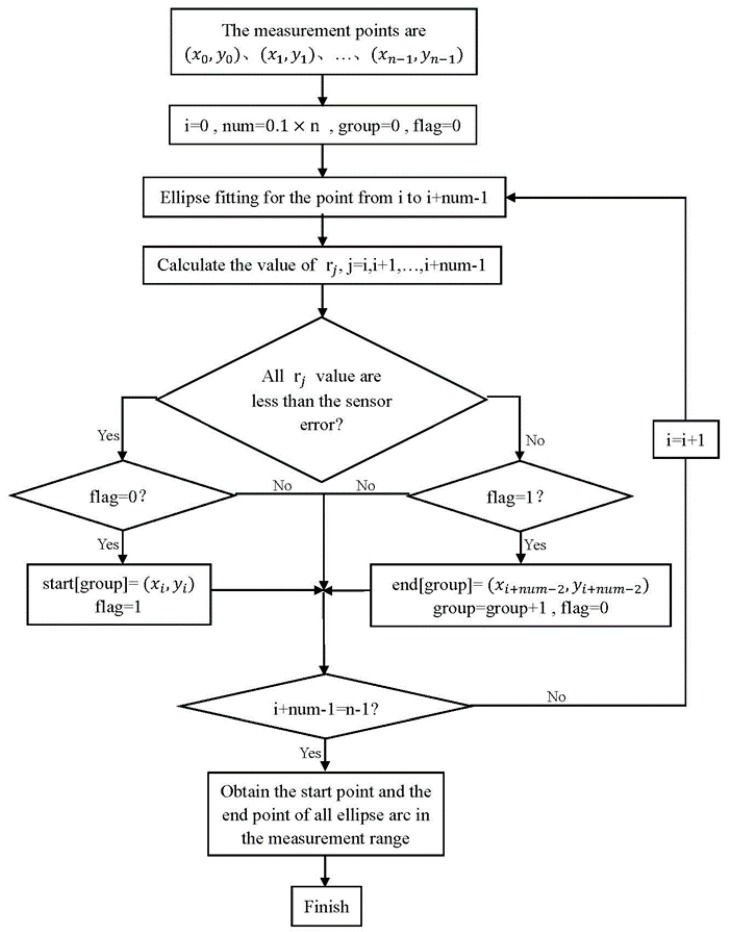
The rough identification method.

There may be one or more cylindrical objects to be measured in one time. The rough identification groups those points by comparing the *r_j_* with the sensor error to determine whether those points can form an ellipse arc. It removes the points that obviously do not belong to the ellipse arc. The effect is shown in Figure 5. The rough identification flow is shown in Figure 6.

**Figure 7:**
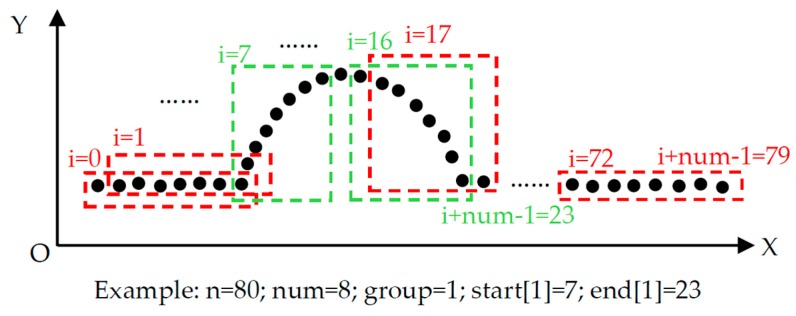
An example of a rough identification method.

The term *n* is the amount of measurement points. The term *num* is the number of points that needs to be fitted each time, which is about 10 percent of *n*. The term *group* records the number of ellipse arcs that have been identified, while *flag* is a mark for indicating that the current point belongs to the start part or the end part. The *start[]* and the *end[]* store the start point and end point of every ellipse arc. The key to rough identification is fitting an ellipse using the points from *i* to *I + num −* 1 in one time, and looping it in turn from *i* = 0 to *I + num −* 1 = *n −* 1. If all *r_j_* (*j* = *i*,… *I + num −* 1) were less than the sensor error and *flag* = 0, the point *i* would be the start point of this ellipse arc. If one of *r_j_* (*j* = *i*,… *I + num −* 1) was larger than the sensor error and *flag* = 1, the point *I + num −* 2 would be the end point of this ellipse arc. The first few points after a start point are the start part. The last few points before an end point are the end part. If all ellipse arcs in the measurement range were obtained, the rough identification would be finished. The method illustrated in Figure 7 is an example of the rough identification process.

#### 3.2.2. Accurate Screening

**Figure 8:**
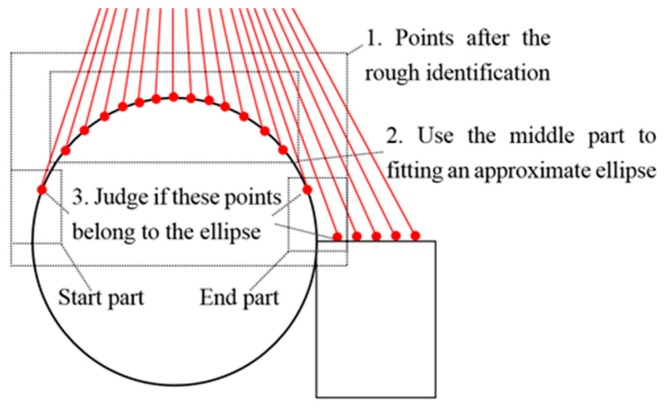
The approach of the accurate screening.

After the rough identification is finished, there are points that may not belong to the ellipse arc. These points lie on the points of the start part and the end part. The accurate screening obtains an approximate ellipse by fitting the points of the middle part, determines which points of the start and end part are on the ellipse, and accurately identifies the points on the ellipse, as shown in Figure 8. Considering the elliptic curve, the *r_j_* of points in the start and end part are calculated. If the *r_j_* was larger than the sensor error, the point would be discarded. As shown in Figure 8, the point in the start part and the first point in the end part will be kept, and the second point in the end part will be discarded.

**Figure 9:**
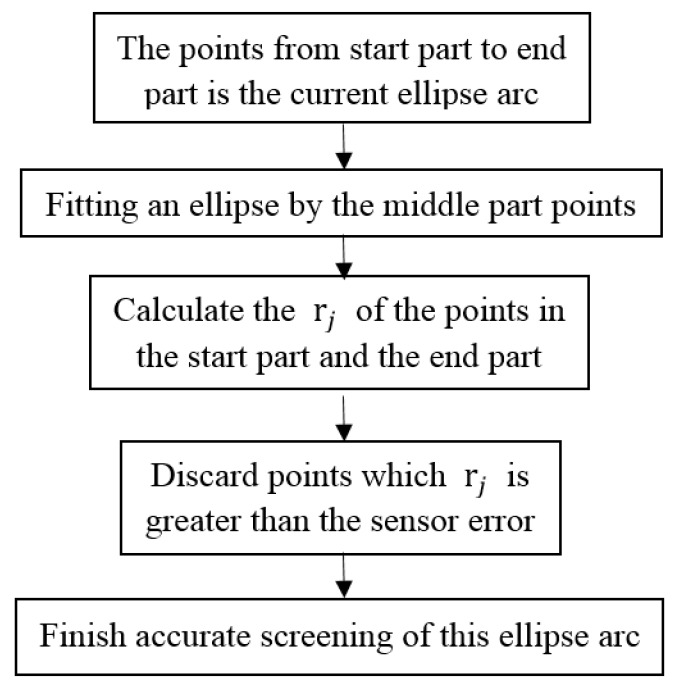
The flow of the accurate screening process.

The flow of the accurate screening is as shown in Figure 9:

### 3.3. Smoothing and Homogenizing

**Figure 10:**
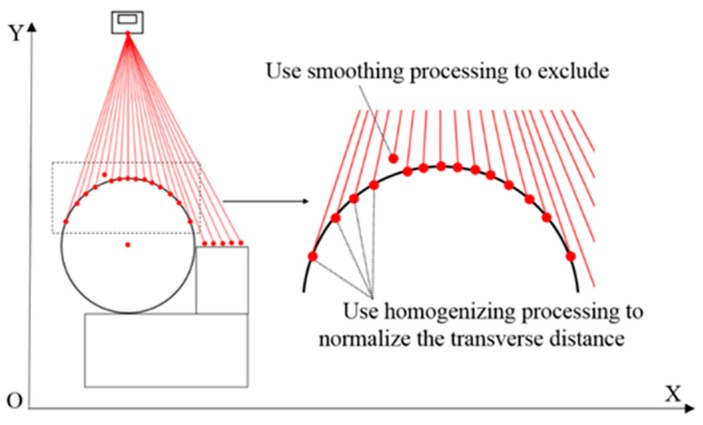
Smoothing and homogenization.

During actual measurement, there are large errors caused by laser line losses or jumps, which must be excluded from the measurement data before fitting, as shown in Figure 10. Because the ellipse recognition involves batch processing of the points and the least squares method is for all the fitting points’ optimal solution, the above ellipse recognition process can’t identify single laser line losses or jumped points effectively. Although the *r_j_* of any lost laser lines and jump points in the ellipse arcs are usually larger than the sensor error, their influence can’t be eliminated by the ellipse recognition process. Based on this, smoothing and homogenization are proposed.

The smoothing and homogenization exclude the large error points and eliminate the influence of variable resolution. The length of the laser in the X direction changes over the distance in the Y direction. The resolution in the X direction decreases with the increasing distance in the Y direction. According to the triangulation measurement principle, the resolution in the Y direction also decreases with the increasing distance. Because the method of fitting uses the least squares method, the uniformity of the points affects the fitting results. It is necessary to normalize the transverse distance to ensure that this influence is reduced to a minimum.

#### 3.3.1. Smoothing

According to the double-smoothing local linear regression method [20], we set the filter window width for 30% of the amount of ellipse arc points. As described in the reference, we need to select the filter window width, and successively select points to filter. We can get the new contour points by the optimal solutions of quadratic polynomial curve in the set of the filter window width.

#### 3.3.2. Homogenizing

Because the highest order of the elliptic curve equation is 2, the data is processed by the cubic spline interpolation in order to avoid the distortion. Assuming that the amount of contour points after the smoothing processing is *n + 1*, the points are record as (*x_o_*, *y_o_*), …, (*x_n_*, *y_n_*).

The piecewise cubic spline curve is as follows:

(6) $$S_{i}\left(x\right)=a_{i}+b_{i}\left(x-x_{i}\right)+c_{i}{\left(x-x_{i}\right)}^{2}+d_{i}{\left(x-x_{i}\right)}^{3}$$

where *i* = 0, 1, 2, …, *n*; *a_i_, b_i_, c_i_, d_i_* are parameters of the cubic spline curve.

There are boundary conditions:

(7) $$\{\begin{array}{l} S_{i}\left(x_{i}\right)=y_{i}\\ S_{i}\left(x_{i+1}\right)=y_{i+1} \end{array}$$

There are continuity conditions:

(8) $$\{\begin{array}{l} {S^{\prime }}_{i}\left(x_{i+1}\right)={S^{\prime }}_{i+1}\left(x_{i+1}\right)\\ {S^{\prime \prime }}_{i}\left(x_{i+1}\right)={S^{\prime \prime }}_{i+1}\left(x_{i+1}\right) \end{array}$$

From Equarions (6)–(8) we have:

(9) $$\{\begin{array}{l} a_{i}=y_{i}\\ b_{i}+c_{i}\left(x_{i+1}-x_{i}\right)+d_{i}{\left(x_{i+1}-x_{i}\right)}^{2}=\frac{y_{i+1}-y_{i}}{x_{i+1}-x_{i}}\\ 2c_{i}\left(x_{i+1}-x_{i}\right)+3d_{i}{\left(x_{i+1}-x_{i}\right)}^{2}=b_{i+1}-b_{i}\\ 3d_{i}\left(x_{i+1}-x_{i}\right)=c_{i+1}-c_{i} \end{array}$$

According to the iterative Equation (9), with the *x* coordinates of the contour points after the smoothing processing, we can get the four parameters of *a_i_, b_i_, c_i_, d_i_* to obtain the formula *S_i_(x)*. After the step interval is set (for example, 0.5 mm), the coordinates *x_new_* predetermined by the step interval between *x_o_* and *x_n_* are obtained. If the *x_new_* was taken into the corresponding piecewise polynomial curve, the *S_i_(x_new_)* would be obtained. The corresponding coordinate *y_new_* is obtained by the polynomial interpolation. Once completing the homogenizing processing of the measurement data, the influence of variable resolution will be eliminated.

## 4. Experiments and Results

**Figure 11:**
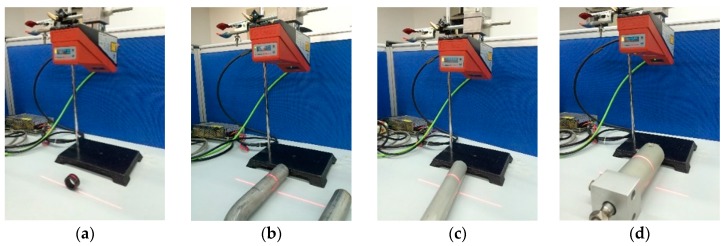
Measurement of single cylindrical objects with radius: (**a**) 15 mm; (**b**) 16 mm; (**c**) 18.5 mm; (**d**) 29 mm.

**Figure 12:**
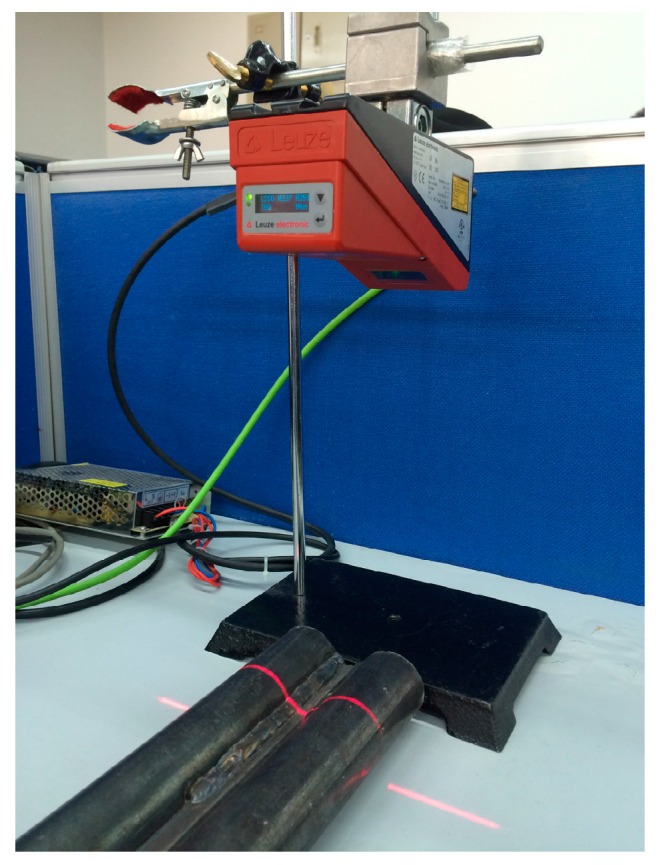
Measurement of cylindrical objects welded together.

**Figure 13:**
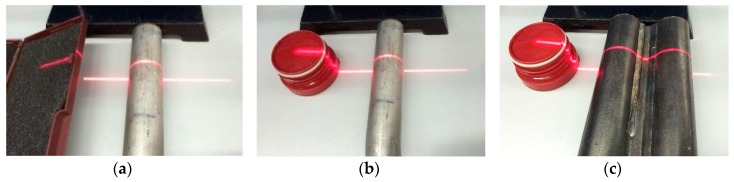
The measurement scene in the case of: (**a**) Single cylindrical object with planar interferences; (**b**) Single cylindrical object with complex interferences; (**c**) Cylindrical objects welded together with complex interferences.

In this section, we describe a series of experiments conducted to verify the correctness of the method and the recognition algorithm, and the robustness of the recognition algorithm. The light section sensor is a LPS36HI unit of Leuze Electronic (In der Braike 1, Owen, Germany). The resolution in the measurement plane is about 0.6 mm × 0.9 mm. The environment for the measuring is an indoor fluorescent lamp. The algorithm is written in Matlab. We employ four cylindrical objects of different radius and put them at different tilt angles to test the measurement accuracy, as shown in Figure 11. Cylindrical objects welded side by side are used to verify the recognition algorithm, as shown in Figure 12. In the presence of several kinds of interference, the cylindrical objects are tested to show the robustness of the recognition algorithm, shown in Figure 13.

### 4.1. Single Cylindrical Object

The measurement results of single cylindrical objects with radius 15 mm, 16 mm, 18.5 mm, 29 mm, the angle *θ* and *φ* 10°, 20°, 30° are shown in Table 1, Table 2 and Table 3.

The experimental results show that the radius measurement errors are within 0.4 mm. Considering the resolution of the sensor is 0.6 mm × 0.9 mm, the measurement is acceptable.

The *θ* value is obtained by fitting the ellipse directly. For different *θ* values, the measurement error is randomly distributed in a 0°~0.2° range. For different *φ* values, the maximum error in 30° is −0.1253°, in 20° is −0.1626°, and in 10° is −0.2140°. The errors of each group are near the maximum values, respectively. This is because *φ* is obtained by calculating a cosine function, and cosine functions are not sensitive to the small angle change values.

This measurement accuracy of the proposed method can meet the requirements of production. If the accuracy of the angle *φ* near the small end of the range were to be improved, it would give a higher resolution sensor.

### 4.2. Multiple Cylindrical Objects

**Figure 14:**
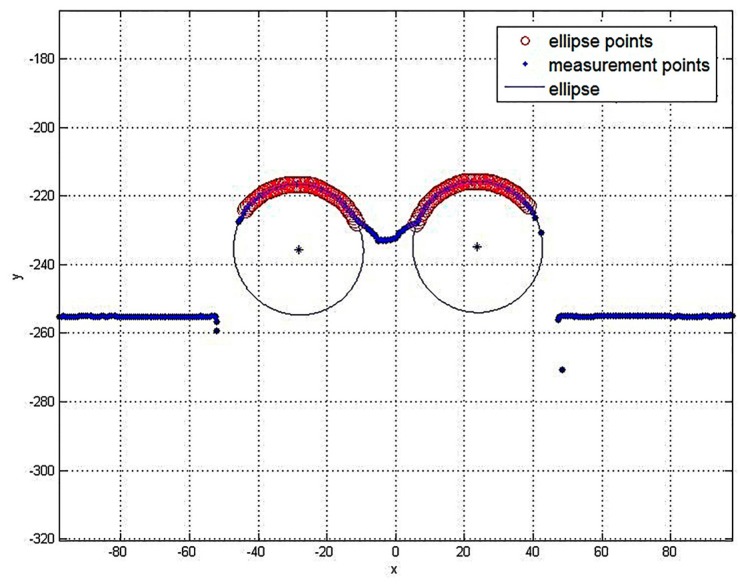
Actual measurement results.

The method can measure multiple cylindrical objects, as shown in Figure 14. The blue dots are the measurement points, the red rings are elliptic feature points, and the blue ellipses represent the cylindrical objects’ section.

In Table 4, each radius is 19 mm. The *θ* varies from 10°, 20° to 30°, and the corresponding *φ* varies from 30°, 20° to 10°.

In Table 4, we can see that the measurement errors show almost the same distribution as those of a single cylindrical object, indicating that the proposed method can precisely measure not only single cylindrical objects, but also multiple cylindrical objects.

### 4.3. Interference

**Figure 15:**
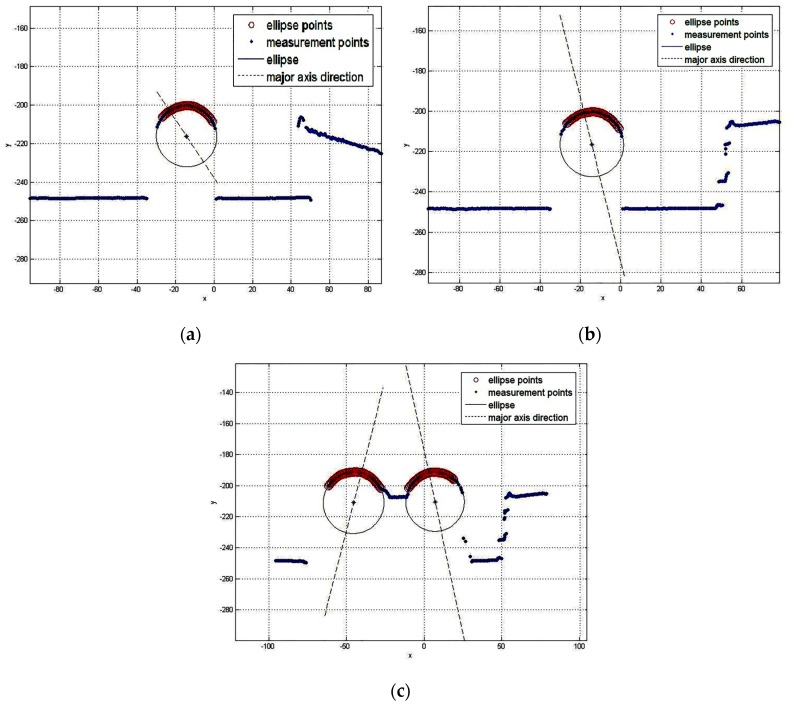
The measurement result in the case of: (**a**) Single cylindrical object with planar interferences; (**b**) Single cylindrical object with complex interferences; (**c**) Cylindrical objects welded together with complex interferences.

In order to verify that the recognition algorithm can identify the ellipse contour points with interferences, we introduce some interference into the measurement. The blue dots are the measurement points. The red rings are the elliptic feature points. The blue ellipses represent the cylindrical objects’ section. The blue dotted lines are the direction of the major axis. Results show that the recognition algorithm can eliminate the interference of arbitrarily shaped objects to accurately identify the ellipse arc from the measurement data, as shown in Figure 15.

**Figure 16:**
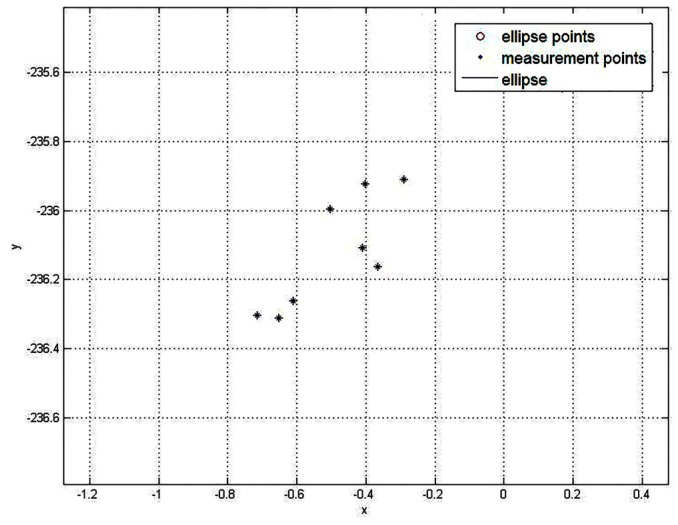
The ellipse center repeatability.

We have measured a cylindrical object many times in the same position, as shown in Figure 16. The ellipse centers can be in a rectangular box of 0.6 mm × 0.6 mm. The ellipse axis errors are less than 0.25 mm. They are within the error of sensor limits.

### 4.4. Discussion

As indicated in the measurement results, the radius errors vary from 0.05 mm~0.4 mm. They are independent of the radii of the cylindrical objects. The *θ* and *φ* errors vary from 0°~0.2°. They are independent of the theoretical angles. We created the routine functions using Matlab, and it took 1~3 s to complete one measurement, as shown in Table 5. The measurement time depends on the number of cylindrical objects measured at the same time and the type of the interference. The more objects that are measured in one time and the more complicated the interferences that exist, the more time is required. 

The surface finish of the cylindrical objects has an influence on the measurement. In order to measure the object, the sensor emits a laser beam and receives the reflected light, so specular surfaces can’t be measured. Even if we change the laser intensity, an excessively high absorbance surface is difficult to measure. Surfaces with good diffuse reflectance achieve the best measurements.

## 5. Conclusions

The main contribution of this paper is the radius and orientation measurement method of cylindrical objects with interference and multi-cylindrical object measurement based on a light section sensor. The method proposed in this paper identifies ellipse arcs by rough identification and accurate screening. After the ellipse recognition is finished, the data are smoothened and homogenized to eliminate the effect of laser line losses or jumps and the influence of variable resolution. The radius and orientation are obtained by fitting the smoothed and homogenized data to an ellipse. The experimental results demonstrate the effectiveness of the proposed radius and orientation measurement method for cylindrical objects.

## Figures and Tables

**Table 1:** The measurement results with *θ* = 10° and *φ* = 30°.

No.	Radius/mm	Radius Error/mm	*θ*/°	*θ* Error/°	*φ*/°	*φ* Error/°
1	14.7543	−0.2457	9.8507	−0.1493	30.0530	0.0530
2	15.8807	−0.1193	10.1130	0.1130	30.1040	0.1040
3	18.3890	−0.1110	10.1706	0.1706	29.9349	−0.0651
4	28.6034	−0.3966	9.8863	−0.1137	29.8747	−0.1253

**Table 2:** The measurement results with *θ* = 20° and *φ* = 20°.

No.	Radius/mm	Radius Error/mm	*θ*/°	*θ* Error/°	*φ*/°	*φ* Error/°
1	14.7369	−0.2631	20.1233	0.1233	20.1270	0.1270
2	15.6270	−0.3730	19.8507	−0.1493	19.8762	−0.1238
3	18.3996	−0.1004	20.1077	0.1077	19.8374	−0.1626
4	28.9285	−0.0715	19.8386	−0.1614	20.1383	0.1383

**Table 3:** The measurement results with *θ* = 30° and *φ* = 10°.

No.	Radius/mm	Radius Error/mm	*θ*/°	*θ* Error/°	*φ*/°	*φ* Error/°
1	14.7740	−0.2260	30.1347	0.1347	10.1887	0.1887
2	15.6107	−0.3893	30.0804	0.0804	10.1678	0.1678
3	18.4960	−0.0040	29.8521	−0.1479	9.7860	−0.2140
4	28.8330	−0.1670	29.8281	−0.1719	10.1884	0.1884

**Table 4:** The measurement results of cylindrical objects welded together.

No.	Radius/mm	Radius Error/mm	*θ*/°	*θ* Error/°	*φ*/°	*φ* Error/°
1	18.9072	−0.0928	10.2417	0.2417	30.1787	0.1787
2	18.9203	−0.0797	9.8943	−0.1057	29.8621	−0.1379
1	18.8611	−0.1389	20.1903	0.1903	19.5594	−0.4406
2	18.9103	−0.0897	19.8831	−0.1169	20.1129	0.1129
1	18.9223	−0.0777	30.2043	0.2043	9.8105	−0.1895
2	18.9360	−0.0640	29.8952	−0.1048	9.7175	−0.2825

**Table 5:** The time of one measurement under different conditions.

Conditions	Time/s	Time per Cylindrical Object/s
Single	1.201	1.201
Welded together	2.526	1.263
Single with planar interferenct	1.343	1.343
Single with complex interferenct	1.471	1.471
Welded together with complex interferences	2.981	1.491
